# Medical News and Miscellany

**Published:** 1899-03

**Authors:** 


### Medical News and Miscellany.

---

**Dr. R. B. Holman** has removed from Dallas to Colorado, Tex.

---

**Dr. C. S. Bobo** has removed from Weatherford, Texas, to Norman, O. T.

---

**Dr. Henry Sappington** (Medical Department University of Texas, 1898,) has located in Austin, Texas.

---

**Doctor,** if you want to sell a good location and practice, correspond with Dr. E. P. Edwards, Paris, Texas. He wants to buy.

---

**Texas** puts on her annual quarantine this year a month earlier than usual, to-wit: April (1st prox.) The Governor has issued his proclamation. All places south of 25° N. latitude are considered infected, and are under the embargo.

---

**Quarantine Matters.**—Dr. J. C. Mayfield, quarantine officer at Galveston, holds over, as he was appointed by Governor Culberson to fill an unexpired term (Blunt) and was reappointed by Mr. Culberson for two years. Governor Sayers appointed at Sabine Pass Dr. B. F. Calhoun, of Beaumont, but he resigned, and Doctor Tackabury has been appointed to the place. Dr. T. lives at Sabine Pass, I think.

---

**The Paper on "Lateral Curvature of the Spine,"** by Dr. A. M. Phelps, published in our February number, was improperly credited to the Transactions of the New York Academy of Medicine. It should have been credited to the Richmond, Va., Academy of Medicine and Surgey, as it was read before that body, and a copy (abstract) was sent us by Prof. Mark Peyser, M. D., the courteous Secretary of that Association.

---

**Texas Eye, Ear, Nose and Throat Charity Hospital, Austin, Texas.**—We have received a copy of the Surgeon's annual report for 1898 to the trustees. This is not a State or county or city hospital, but is a subscription and self-sustaining institution under the patronage of Austin ladies, and Dr. Hilgartner, the executive surgeon, gives his services free. The increase of cases treated over the previous year was 16 per cent.

---

**How to Secure Safe and Successful Vaccination.**

---

Several methods may be used in vaccinating; the following usually insures good results: With soap and water thoroughly cleanse the site of inoculation, then with a pledget of cotton dipped in alcohol rub the spot. Allow the alcohol to completely evaporate. Break off one end of the vaccine tube and insert it in the rubber bulb; then with a sterile scalpel or needle scarify the site of inoculation in the usual manner. It is possibly better not to draw blood, but a little will do no harm. Be sure to scarify deeply enough for serum to freely exude. Now break off the other end of the vaccine tube. Place the thumb over the hole in the rubber bulb and eject the lymph on the scarified area, then rub it in *thoroughly* with the instrument used for the scarification. Glycerinated vaccine is not as readily absorbed as dry limph; glycerin is hygroscopic and tends to attract the serum from the tissues to itself. *Do not bandage the arm or pull down the sleeve until the lymph is dry.*

